# Health Damage of Air Pollution, Governance Uncertainty and Economic Growth

**DOI:** 10.3390/ijerph20043036

**Published:** 2023-02-09

**Authors:** Yi Zhang, Mengyang Wang, Tao Shi, Huan Huang, Qi Huang

**Affiliations:** 1 School of Business, Jiangsu Normal University, Shanghai Road 101, Xuzhou 221116, China; 2 School of Government, Sun Yat-sen University, Xingangxi Road 135, Guangzhou 510006, China; 3 Economics Institute, Henan Academy of Social Science, Gongxiu Road 16, Zhengzhou 451464, China; 4 Hebi High-Quality Development Research Institute, Jiangdong Road 1, Hebi 458030, China; 5 School of Business, Chengdu University of Technology, Digital Hu’s Line Research Institute, Chengdu University of Technology, Dongsan Road 1, Chengdu 610059, China; 6 Zhengzhou Central Sub-Branch of People’s Bank of China, Shangwu Road 21, Zhengzhou 450000, China

**Keywords:** health damage of air pollution (APHD), uncertainty of governance, economic growth, moderating effect, threshold effect

## Abstract

The evaluation of environmental and health governance processes is an important part of the innovation and perfection of modern governance systems. Based on the macropanel samples, this paper analyzes the impact of the health damage caused by air pollution (APHD) on economic growth and the related mechanisms accordingly using the moderate model and the threshold model. The results can be concluded as follows: (1) After locking in the health damage perspective, the APHD has a negative impact on economic growth. When other conditions are met, economic growth will significantly drop by 1.233 percent for each unit increase in the APHD index. (2) There is a moderate effect of governance uncertainty in APHD on economic growth with different characteristics. The combination of governance uncertainty and APHD can significantly inhibit economic growth, and this moderating effect has different impacts due to heterogeneous conditions. Spatially, this inhibitory effect is significantly obvious in the eastern, central, and western regions, while the negative effect is significant in areas north of the Huai River with medium and low self-defense ability. Additionally, compared with the delegating of governance power at the municipal level, when the governance power is delegated at the county level, the interaction between the governance uncertainty constructed by income fiscal decentralization and APHD has a less negative economic effect. (3) There is a threshold effect under the conditions of a low level of decentralization of prevention and control, a high level of investment in governance, and a low level of APHD. However, under the condition of a certain APHD level, when the decentralization level of pollution control is higher than 7.916 and the input level of pollution control in GDP is lower than 1.77%, the negative moderating effect can be effectively reduced.

## 1. Introduction

The sound development of environment and health is an important factor affecting the regional economy to promote high-quality development. The extensive economic growth mode, accompanied by rapid industrialization and urbanization, has directly led to China’s total air pollution emissions far exceeding those of other countries [1]. The aggravation of air pollution has become an increasingly serious challenge to China’s public health, social welfare, and economic growth [2,3]. Particularly, particulate matter and ozone pollution can cause China’s economy to lose up to 5% of its GDP [4]. Air pollution has also reduced the life expectancy of residents in the north of the Huai River by an average of five years compared with that in the south [5]. China has actively taken action to address the health problems of air pollution, mainly through three stages: coal control, sulfur control, and comprehensive governance, following the three main sources of China’s air pollution: soot type, automobile exhaust type, and mixed type pollution [6]. The governance mode has also been dominated by “command and control” administrative leadership by the government. However, China’s air pollution control intensity has gradually strengthened recently, but the growth rate of the cost-benefit ratio is not so high. For example, since the State Council issued the Interim Measures for the Collection of Pollution Discharge Fees in 1982, the Chinese government issued the Notice on the Pilot Work of Levying Sulfur Dioxide Pollution Discharge Fees from Industrial Coal Fires in 1992. In 2017, more than 70% of China’s cities still failed to achieve air quality standards [6]. This phenomenon shows that the Chinese government’s input cost/benefit ratio for air pollution control is still low. The main reason may be that China has an obvious system of decentralized governance. In the process of implementing governance policies, different administrative entities are prone to “hitchhiking,” which makes it difficult to maintain collaborative governance and causes the serious problems of non-collaboration [7]. However, this non-synergy is due to the existence of such factors as the political ranking system [8], the division of territorial governance [9], fiscal decentralization and other fragmentation of property rights [10,11,12], and the inequality in economic growth [12], which lead to the insufficient and uncoordinated use of governance rights and lack the efficient joint defense and governance mechanism.

The synergy of pollution control is an important factor affecting pollution control. Although China has achieved coordinated governance in special periods and effectively improved air pollution [13,14]. However, authors generally believe that the current governance synergy in China is still insufficient. This naturally aggravates the uncertainty of governance, which mostly manifests in the fact that environmental governance policy may lead to multiple results due to improper implementation [9,12]. This uncertainty is not only reflected in the choice between environmental improvement and economic growth but also in the impact of environmental improvement or economic growth. The implementation of environmental policies did not significantly improve the overall relationship between China’s extensive economic growth, which increased environmental pollution, and environmental pollution inhibiting economic growth, mainly because environmental policies were not effectively implemented [15]. Although China has made some achievements in environmental and health governance since 2016, the governmental input cost caused by governance policies and the impact on economic growth in the governance process cannot be ignored. On 3 March 2020, the issuance of the Guiding Opinions on Building a Modern Environmental Governance System highlighted the urgency of innovating and improving the environmental governance system. 

Furthermore, combining with the views of traditional economists, environmental pollution has the feature of null jointness, which is the product of extensive economic growth [16,17]. This shows that serious environmental pollution is often accompanied by high-level economic growth, but it will also have damaging health effects that hinder economic growth. The relevant governance policies issued by the state are often accompanied by environmental governance policies and health governance policies. The ultimate goal is to eliminate the health hazards caused by ecological pollution, especially air pollution. The health hazards caused by air pollution are more universal and widespread (as pointed out in the Lancet’s 2010 Global Disease Burden Assessment report, outdoor air pollution has become the fourth most lethal risk factor in China). Therefore, we consider the health damage effect, measure the index of air pollution-health damage (APHD), and construct the uncertainty index of the governance process based on the perspective of assessment of the governance process at the macro level to analyze the relationship between the APHD, governance uncertainty, and economic growth. The marginal contributions of the article are as follows: (1) Locking the health damage effect of pollution, the APHD has a negative impact on economic growth under a more accurate assessment of the APHD. (2) Based on the reality of China’s decentralized prevention and control system, the governance uncertainty index is measured from the perspective of the policy implementation process, which is beneficial for others to learn from and construct panel data for research. (3) The relationship among APHD, governance uncertainty, and economic growth has been studied. We found that governance uncertainty has a moderating effect on the negative economic effects of APHD. 

The following sections are as follows: (1) The second part is the literature review and research questions; (2) The third part is the research design and data description; (3) The fourth part is the empirical results analysis; (4) The fifth part is the discussion; (5) The last part is the conclusion and policy implications.

## 2. Literature Review

### 2.1. The Impact of APHD on Economic Growth

The impact of APHD on economic growth can be traced back to research on the relationship between environmental pollution and economic growth. Some scholars mainly discuss the relationship between environmental pollution and economic growth within the framework of the environmental Kuznets curve (EKC) and believe that the relationship between environmental pollution and economic growth is in an inverted U shape [18,19] or other types (e.g., an N shape [20]). This result shows that environmental pollution has different effects on economic growth under different conditions. This is mainly because there are many ways for environmental pollution to affect economic growth. As a result, some scholars jumped out of the category of EKC theory and explored the relationship between them from the perspective of multiple specific mechanisms: First, the mechanism of environmental production factors: pollution damages the stock or flow of productive substances, thereby hindering economic growth [21,22]. Second, the mechanism of capital accumulation: pollution will damage the health of residents, increase the medical burden on families, squeeze out physical investment (savings), hinder capital accumulation, reduce capital flows to enterprises, and affect economic growth [23]. At present, some scholars focus more on the economic burden effect caused by APHD [24], and the external cost of air pollution in China accounts for 1−8% of GDP [25]. Other scholars have directly studied the impact on health care expenditure [26,27,28], and every 1% increase in the proportion exposed to PM2.5 would increase household medical expenditure by 2.942% [28]. Studies above have potentially shown that air pollution will damage health, increase household burdens, and have a negative impact on the economy. However, there are few empirical studies directly discussing the relationship between APHD and economic growth. 

Third, the functioning mechanism of the labor market: the labor market effect of air pollution on economic growth mainly includes the direct effect path of labor supply and the indirect effect path of labor emotion. (1) The direct path of labor supply: air pollution will lead to illness or death of workers, causing absenteeism [29], loss of working days [30,31], and reduction of life expectancy [5], which directly reduce the total labor hours and work efficiency [32,33]. For example, excluding the exogenous impact of a refinery closure event in Mexico City, according to the SO_2_ concentration measurement, the refinery closure led to a 19.7% decrease in the surrounding pollution level, which led to a 3.5% increase in workers’ working hours per week [31]. This means that air pollution harms public health, reduces the total labor supply level, and also has a negative impact on the economy. (2) The indirect path of labor emotion. Air pollution affects workers’ psychological emotions, leading to changes in their investment decisions [34,35] or employment decisions [36,37]. For example, air pollution has triggered the pessimism of investors, which has a negative impact on the return, liquidity, and volatility of regional enterprises’ stocks, further affecting regional economic growth [35]. Therefore, we can see that air pollution damages public health, and this negative impact can play a significant role in the labor market, which will hinder regional economic growth. 

### 2.2. The Impact of Governance Uncertainty on Economic Growth

Governance uncertainty mainly comes from the implementation of policies. At present, most scholars have studied the economic effects brought by the uncertainty of economic policy, monetary policy, and fiscal policy [38,39]. Among them, some scholars believed that the fiscal policy uncertainty has a positive impact on economic growth [40,41], a negative impact [39], or no impact [42,43]. However, few studies on the economic effects of the governance uncertainty originated from the implementation of environmental policyies. The implementation of environmental policies has two main effects on economic growth: the first is the impact of the policy itself on economic growth, and the second is the impact of policy implementation on economic growth. 

The research on policy itself shows that there are uncertainties in the impact of different environmental policies on the economy. A single type of environmental policy, such as an environmental tax (pollution tax), a labor tax, an energy tax, etc., can have a positive or negative impact on economic growth by affecting income redistribution, adjusting employment, changing consumer demand, etc. [44,45,46,47,48]. Additionally, the input policy of environmental pollution control also has an uncertain impact on economic growth [49,50]. Environmental governance investment is conducive to long-term economic growth but not to short-term economic growth [50]. It may also be conducive to short-term economic growth but has no impact on long-term economic growth [51], etc., while comprehensive environmental regulation policies have positive and negative uncertain impacts on economic growth [52,53]. 

Further, the impact of policy implementation on economic growth is studied. Some scholars confirmed that policy formulation and implementation should be tailored to local conditions to ensure economic growth [37,54]. For example, it is necessary to coordinate regional policies and regulations on environmental protection and employment promotion and reasonably provide a pollution subsidy for government and enterprises to improve the willingness of talents to work in polluted cities, ensuring the development of the local economy [37]. However, there are a few quantitative studies on this. The implementation of environmental policies did not significantly improve the current situation of environmental pollution inhibiting economic growth because China’s environmental policies were not fully implemented [15]. Additionally, most studies focus on the effect of the mode of implementation of environmental policies. Scholars generally believe that the decentralized prevention and control mode in China is prone to produce environmental “race to bottom” effects and GDP catch-up effects [9,12]. This indicates that the improper implementation of environmental policies will bring uncertainty, which has an uncertain impact on economic growth. Some scholars, from the perspective of general policy implementation, found that the impact of fiscal decentralization on economic growth is uncertain and depends on the impact of other systems, such as tax decentralization and administrative decentralization, etc. [55,56]. 

Therefore, we can see that the governance uncertainty mainly comes from the implementation of the policy itself and the way in which policy is implemented. The impact of policy itself can be measured by the cost of policy implementation input, and the influence of policy implementation mode is mainly reflected through the decentralization governance mode. Decentralized governance (or decentralized implementation) not only includes fiscal decentralization but also the decentralization of economic development and administrative jurisdiction. Few studies consider the decentralization mode of governance from a comprehensive perspective, and especially few consider both the impact of policy itself and the way of policy implementation and study its economic growth effect from the perspective of governance uncertainty accordingly.

### 2.3. APHD, Governance Uncertainty, and Economic Growth

Currently, few references have studied the relationship between APHD, governance uncertainty, and economic growth. Some scholars believe that the strict implementation of relevant APHD policies can promote economic growth from an overall governance perspective [57,58,59]. Other scholars believe that the implementation of relevant APHD policies will affect investment decisions in heavily polluted areas and hinder local economic growth [60]. Further from the perspective of joint prevention and control of governance, scholars believe that joint prevention and control policies can effectively alleviate the negative effects of pollution on economic growth, although air pollution inhibits economic growth [61,62]. Joint Regional Air Pollution Prevention and Control System (JPCSRAP) can achieve a win-win outcome for both the environment and the economy, namely reducing air pollution while achieving economic growth [62]. 

**Figure 1:**
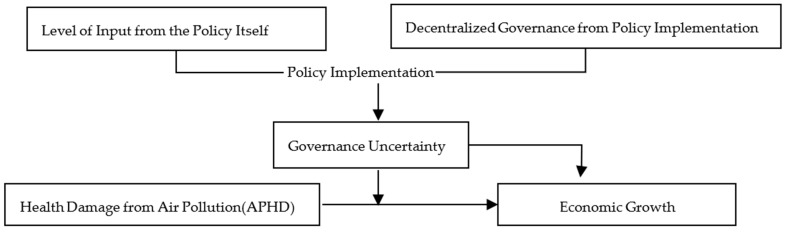
The theoretical framework.

Above, previous studies have provided a good research basis for us. However, currently, scholars have no consensus on the relationship between environmental pollution and economic growth because of the different ways that environmental pollution affects economic growth and the impact on health damage. Meanwhile, most studies on the relationship between environmental policies and economic growth only focus on the policy itself without paying attention to the policy implementation process, and few attribute the uncertain results brought by such policies to the process of policy implementation. Therefore, it needs to further verify the relationship between health damage caused by pollution and economic growth, especially the moderate effect of governance uncertainty between them. There are few references to constructing the governance uncertainty index based on different governance methods and governance investment in the governance process. From above, the assessment framework is shown in Figure 1, and the three main questions are as follows: (1) Does air pollution’s health damage hinder economic growth? (2) Will governance uncertainty further aggravate the negative impact of air pollution on health damage and have a moderate effect on economic growth? And is the moderating effect affected by different endowment conditions, especially under heterogeneous conditions? (3) Is there a threshold effect for the regulatory effect of APHD on economic growth under the conditions of environmental governance investment, decentralization level of prevention and control, and health damage caused by air pollution? 

## 3. Materials and Methods

### 3.1. The Econometric Model

Firstly, we analyze whether the interaction between APHD and governance uncertainty has an impact on economic growth, and the benchmark model is as follows:

(1) $$lny_{\mathrm{it}}=\beta_{0}+\beta_{1}h_{it}+B\times contr+\omega_{i}+\delta_{t}+\varepsilon_{it}$$

(2) $$lny_{\mathrm{it}}=\beta_{0}+\beta_{1}h_{it}+\beta_{2}ui_{it}+\beta_{3}h_{it}\times ui_{it}+B\times contr+\omega_{i}+\delta_{t}+\varepsilon_{it}$$

In Equations (1) and (2), *i* and *t* refer to the province and the year, respectively. The value y represents the economic level, and h represents the coupling index of air pollution and health damage (APHD). $\beta_{1}$ refers to the economic growth rate changing when h changes one unit. *ui* denotes the governance uncertainty index, *h × ui* is the interaction term, and ω, δ, and ε refer to the individual effect, the time effect, and the random effect, respectively. B represents the coefficient parameter vector of the control variables, $\beta_{0}$ is the constants, $\beta_{2}$, and $\beta_{3}$ represent the coefficient parameters, and *contr* represents the control variables.

Secondly, we adopt the threshold effect model to further explore at what level the APHD influences economic growth. Meanwhile, we decompose the governance uncertainty into two aspects: the input level from the policy itself and the decentralized governance level from the policy implementation process, and further explore to what level they affect economic growth. The model is as follows:

(3) $$lny_{\mathrm{it}}=\mu_{0}+\mu \times M\times I\left(\cdot \right)+\omega_{i}+\delta_{t}+\varepsilon_{it}$$

In Equation (3), $\mu_{0}$ refers to the constant, μ refers to the coefficient, and *M* is the vector set of explanatory variables. *I (·)* represents the indicative functions of each threshold condition. The conditions in the *I (·)* include APHD, the input level from the policy itself, and the decentralized governance level from the policy implementation process.

### 3.2. The Variable Measurement

#### 3.2.1. The Dependent Variables

**Figure 2:**
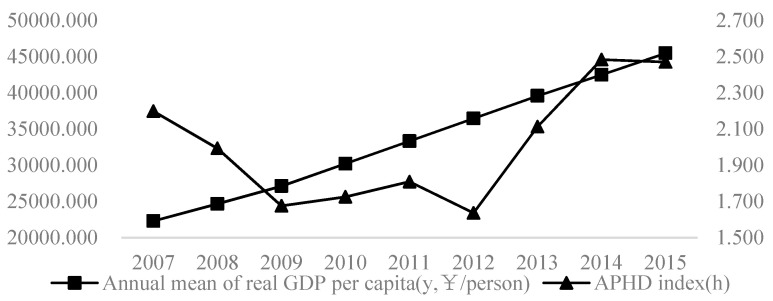
Average change of economic growth and APHD during the study period.

The dependent variable is economic development level (*y*). We use the natural logarithm of GDP per capita to represent regional economic levels [46]. The changing trend is shown in Figure 2.

#### 3.2.2. The Explanatory Variables

The first main independent variable is the level of health damage caused by air pollution (APHD, *h*). Scholars of environmental epidemiology and environmental toxicology believe that there is a reaction relationship between air pollution and multiple health endpoints [5,63,64]. However, most of the current studies involving the issues of environmental health economics often use the concentration of one or more air pollutants to directly represent the connotation of APHD and explore its economic effects, ignoring the consideration of health damage [65]. However, since 2010, APHD has become an important factor affecting the normal economic life of Chinese [66]. 

Meanwhile, traditional economics believes that environmental pollution is characterized by null jointness, which is the product of extensive economic growth; that is, serious environmental pollution is often accompanied by high-level economic growth, and the null-jointness effect is also suited to air pollution. In order to avoid the single impact of air pollution on economic growth, this paper chooses the coupling index *h* of the air pollution system and the health damage system (APHD) to analyze, considering the health damage effect. Further, in order to fit the development strategy of promoting the construction of “healthy China” and highlight the important role of healthy human capital, the comprehensive index system and principal component entropy weight method are adopted to measure the APHD index (*h*) [66]. Moreover, Figure 2 shows the development trend of APHD calculated by the annual mean value of health damage estimated by air pollution from 2007 to 2015. In Figure 2, there is a fluctuating trend of decline first and then increase from 2007 to 2015 in China. Among them, the rise of APHD before 2014 was mainly due to the rapid growth rate of APHD in eastern China. However, after 2014, the decline of APHD was due to the decline in western China.

The second independent variable is the Governance Uncertainty Index (*ui*). The definitions of *bp* and *bpp* in Table 1 are used to represent two uncertainty indices, respectively. Current research on governance uncertainty mainly involves economic policy uncertainty, fiscal policy uncertainty, and so on. Most of them use the uncertain words and texts involved in policiy introductions for measurement. For example, the economic policy uncertainty index mainly includes three quantitative components: newspaper reports on policy-related economic uncertainty, the number of federal tax law provisions that will expire in the next few years, and the differences among economic forecasters [67]. Although the measured uncertainty index is comprehensive, scientific, and widely applied, it has two limitations: First, the calculation for China is not specific to the provincial level or smaller level, so scholars cannot use panel samples for analysis; second, it only focuses on the measurement of the uncertainty of economic and fiscal policies and lacks the measurement of the uncertainty of environmental governance policies. Therefore, this paper innovatively designs and measures the uncertainty of environmental policy implementation with panel change characteristics. In the process of policy implementation, there are two main sources of governance uncertainty: First, the size of investment in governance—under the decentralized governance system, pollution control investment has the dual purpose of economic growth and environmental improvement. This is often closely linked to rent-seeking behavior by officials, which can affect development [68,69,70]. Therefore, this paper believes that the greater the input costs and the more elastic the space to bring rent-seeking behavior, the greater the uncertainty of development. The second is the implementation process of decentralized prevention and control. There is a symbiotic relationship between decentralized and democratic governance [71]. Therefore, decentralized prevention and control is often accompanied by multiple governance outcomes, such as the environmental “race to the bottom” effect, the GDP catch-up effect, and the anti-commons tragedy effect [9,12]. This will exacerbate uncertainty about governance outcomes. To sum up, the level of decentralization of prevention and control determines the level at which each subject can achieve the “same effort” in pollution control, that is, the greater the level of decentralization of prevention and control, the greater the possibility of governance uncertainty [12]. Therefore, the decentralization level of pollution prevention and control in each region and each year can be regarded as the weight of pollution control input, and the product of the two can be regarded as a comprehensive index to measure the uncertainty of pollution control, which can be expressed by the following formula:

(4) $$bp_{it}=proex_{it}\times bxt1_{it}$$

(5) $$bpp_{it}=proex_{it}\times bxt2_{it}$$

Among them, *bp* and *bpp* both represent the uncertainty of governance. The difference between them is that they are endowed with weights expressed by different levels of prevention and control decentralization. They can be regarded as proxy variables for robustness tests. *proex* represents the amount of investment in governance; *bxt1* and *bxt2* refer to the decentralization level of prevention and control delegated to the municipal level and the county level, respectively. The decentralization types of pollution control include fiscal decentralization, territorial jurisdiction decentralization, and economic decentralization [12]. The level of decentralized governance discussed in this paper is the comprehensive level of decentralized governance, namely:

(6) $$bxt1_{it}=\frac{1}{3}efd_{it}\times \frac{1}{3}citj_{it}\times \frac{1}{3}ged_{it}$$

(7) $$bxt2_{it}=\frac{1}{3}rfd_{it}\times \frac{1}{3}cotj_{it}\times \frac{1}{3}red_{it}$$

**Figure 3:**
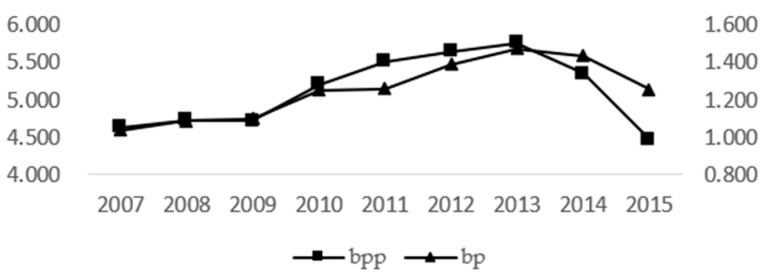
Changes in the two governance uncertainty indices during the study period.

Above, *efd* represents expenditure-oriented fiscal decentralization, and *rfd* represents revenue-oriented fiscal decentralization. *citj* and *cotj* represent the decentralization of territorial jurisdiction delegated to the municipal level and the county level, respectively. *ged* refers the economic decentralization measured by the Gini coefficient method, and *red* stands for economic decentralization measured by the range method [12]. As shown in Figure 3, according to the measured changes of the national mean, the uncertainty of governance was always on the rise before 2013 and began to decline after 2013. That is, after the implementation of the Action Plan for Air Pollution Prevention and Control in 2013, the Yangtze River Delta region took the lead in launching the cooperation mechanism for air pollution prevention and control. It shows the development trend of domestic joint prevention and control, which reduces the uncertainty of pollution control and contributes to pollution prevention and control. For example, in 62 Chinese cities tracked by the WHO, particulate pollution decreased by an average of 30 percent between 2013 and 2016.

The third independent variable is uncertainty in the implementation of policies related to APHD. We used the interaction term between the governance uncertainty index and the APHD index to represent its connotation. We respectively used *hbp* and *hbpp* to represent the interaction terms between the above two governance uncertainty indices, and the APHD index to represent the uncertainty caused by the implementation of relevant governance policies.

According to the selection of benchmark model variables, the threshold variable mainly includes the APHD index (*h*), the amount of investment in governance (*proex*), and the decentralization level of prevention and control delegated to the county level (*btx2*) [9,12]. These are the main factors affecting uncertainty. Here, *btx2* is used to participate in the analysis, mainly for the following reasons: First, the selected threshold variables are required to contain richer change information, and the change information of the prevention and control decentralization variables that are delegated to the county level is more sensitive, while the prevention and control decentralization variables that are delegated to the municipal level are relatively less in each province, and the change is rigid. Second, many studies have shown that decentralization of governance power has a more significant impact on economic growth and air pollution-related governance effects [72,73,74]. Selecting *btx2* to participate in the analysis can more accurately identify whether there is a threshold effect. Therefore, the threshold effect analysis is mainly carried out by using the governance uncertainty index (*bpp*) constructed by *btx2*.

#### 3.2.3. The Control Variables

Set of control variables (*contr*): According to research, per capita capital stock (*k*), regional openness (*iemport*), human capital level (*pere*), the ratio of secondary industrial structure (*secr*), urbanization level (*ur*), and other variables are mainly chosen [72,73]. Detailed calculations and definitions of the above variables are shown in Table 1 below.

### 3.3. The Sources of Materials

The data used in this article are from provincial statistical yearbooks in China, the China Stock Market and Accounting Research Database (CSMAR), the China Economic Network Statistical Database, the China Statistical Yearbook, the China Health Statistics Yearbook, the China Environmental Statistics Yearbook, and the meteorological monitoring data of Columbia University, etc. We set the study period as 2007–2015. The reasons for selecting this sample period are as follows: First, some indicators involved in the measurement of the APHD index were only available in the sample period up to 2015, and most of the different health endpoints involved statistically started in 2007. Second, the governance uncertainty index constructed in this paper has a lot to do with the division of governance rights. Since 2013, China has begun to further promote joint defense and governance. Although this interfered with the influence of administrative jurisdiction judgments to some extent, it was not until 2016 and later that the Yangtze River Delta and other regions gradually and comprehensively implemented joint defense and governance. At the same time, in 2016, China began to implement the healthy China 2030 Development Strategy. This means that the relevant exogenous policies will have less interference before 2015. The data on the value of money in the sample are price adjusted for the 2007 base period. In addition, the depreciation rate of different provinces varies greatly and cannot be obtained directly [6]. Meanwhile, the perpetual inventory method was used to calculate the capital stock [75]. See Table 1 for details.

## 4. Results

### 4.1. The Baseline Results

First, using a Hausman test, the results show that the fixed effect model should be selected for analysis. A VIF value less than 10 indicates that there is no multicollinearity. The variables related to interaction items in the model have been decentralized. The mean (*) in marginal effect analysis represents the mean value of the variable “*”.

As shown in Table 2, APHD has a significant negative impact on economic growth, and governance uncertainty has a positive impact on economic growth. However, the interaction between governance uncertainty and APHD has a significant negative impact on economic growth. These results suggest that the uncertainty in the implementation of policies to address issues related to APHD in China has an inhibitory effect on economic growth. This is consistent with previous studies. They believed that the implementation of environment-related policies would have a negative impact on economic growth in the short term [50,53]. From the results of stepwise regression estimation, the overall estimation in Column (1) ~ (9) is relatively robust. Specifically, as shown in Column (9), when other conditions are given, for each one-unit increase in the APHD index, economic growth will significantly drop by 1.233% at a 1% confidence level. The calculation formula is: $100\times \left(\beta_{1}+\beta_{3}\times mean\left(bp\right)\right)\%\approx -1.233\%$.The coefficient of the governance uncertainty is significantly positive at the 5% confidence level, indicating that economic growth will increase by 1.174%, with a 1% increase in the governance uncertainty. The calculation formula is: $100\times \left(\beta_{2}+\beta_{3}\times mean\left(h\right)\right)\%\approx 1.174\%$. This is basically consistent with previous research views. For example, the promoting effect of fiscal decentralization on economic growth depends heavily on the authority of local governments, and tax decentralization will lead to higher (or lower) economic growth rates when combined with high (or low) administrative and political decentralization [56]. Some scholars further confirmed that fiscal decentralization can promote local economic growth under certain conditions [72,73]. In this paper, governance uncertainty is measured based on the level of decentralization prevention and control of independent governance. The connotation of power not only contains the connotation of fiscal decentralization but also includes the connotation of economic decentralization (of the same origin as tax decentralization) and administrative decentralization, which is a comprehensive connotation of comprehensive decentralization prevention and control. The governance uncertainty has a positive effect on economic growth, which accords with the previous research viewpoint. However, from the perspective of interaction terms, the governance uncertainty will exacerbate the inhibiting effect of APHD on economic growth. Other things being equal, economic growth will fall by an average of 1.3% for every unit increase in the interaction term. In Column (9), when controlling other conditions, for each 1% increase in the interaction item between APHD and governance uncertainty, economic growth will significantly drop by 1.3% at the 1% confidence level. The governance of issues related to APHD relies on decentralized governance, which is not conducive to collaborative governance, and the uncertainty generated will have a negative impact on economic growth through the implementation of relevant governance policies [15,37,54].

### 4.2. The Robustness Test

We adopt three main ways to test the robustness of the results above, as shown in Table 3. First, we remeasured the governance uncertainty index. Based on the dissonance of policy implementation, individual scholars delegated the power of territorial governance on the county level to measure the fiscal power from the perspective of fiscal revenue and the division of economic development rights using the range method and recalculated the dissonance index of governance implementation [12]. Based on this, we recalculated the decentralization level of governance and took it as a new weight to construct the proxy variable (*bpp*) of governance uncertainty and its interaction term (*hbpp*) for testing. The result is shown in Column (10). Second, in order to overcome the endogeneity between APHD and economic growth, we used the lag period of APHD (*L.h*) as the proxy variable of the current period to construct an interaction term (*L.hbp*) for testing. The result is shown in Column (11). Third, we added the proxy variable of APHD (*L.h*) and the newly calculated proxy variable of governance uncertainty (*bpp*) into the model at the same time to construct interaction terms (*L.hbp*) for testing. The result is shown in Column (12). 

As shown in Table 3, both the APHD of the current period and the delayed one significantly inhibit economic growth, and the difference between these impacts is small, as shown in Column (10)~(11). The interaction terms of APHD and governance uncertainty both significantly inhibit economic growth. However, due to the different measurement levels of governance uncertainty, the impact is quite different. Specifically, the governance power is delegated to the municipal level, and the interaction between the governance uncertainty constructed by expenditure fiscal decentralization and APHD has a clearly negative economic effect. As shown in Column (11), the average elasticity of impact is about 1.3%. While the governance power is delegated to the county level, the interaction between the governance uncertainty constructed by income fiscal decentralization and APHD has a small negative economic effect, with the average elasticity of influence ranging from 0.2% to 0.3%, as shown in Column (10) and (12). Delegating environmental governance power to the county level is more conducive to environmental governance and economic growth than delegating power to the city level [76]. Meanwhile, income fiscal decentralization automatically matches the economic capacity of each region and is more efficient in the process of environmental governance. This result confirms the view that income fiscal decentralization has better effects on environmental governance than expenditure fiscal decentralization [12]. 

### 4.3. Results of Regional Conditions

Furthermore, from a regional perspective, we analyzed the impact of the interaction between governance uncertainty and APHD on economic growth. As shown in Table 4, according to Column (13)~(15), the interaction terms of governance uncertainty and APHD in the eastern, western, and eastern regions can significantly inhibit economic growth, but the impact is larger in the western region. When other conditions remain unchanged, for every unit of the interaction term that increases, the economic growth in the western region significantly drops by 7.9%. In the four regions mentioned above, the APHD level in the western region is the lowest, with an average of 1793, but its governance investment is the largest, with an average of 1.651, while the governance uncertainty is relatively large, with an average value of 1145. Therefore, the interaction term in the western region has a larger impact on economic growth. These results indicate that there is a mismatch between the environmental governance input and the APHD level in the western region, which increases the governance uncertainty and leads to the interaction terms significantly inhibiting economic growth and sacrificing greater economic benefits. Similarly, the governance input of the central region is greater than that of the eastern region. Under the decentralized prevention and control system, the governance uncertainty of the central region is greater than that of the eastern region, so the negative impact on its economic growth is greater than that of the eastern region. 

According to the Huai River boundary (see Column (16)~(17)), the interaction terms of governance uncertainty and APHD in the north of the Huai River significantly inhibit economic growth, with an inhibition elasticity of 1.6%. Although the study showed that the APHD was more serious in the north of Huai River [77], which was mainly caused by the indoor heating policy in the north, the results of this paper, which mainly used outdoor indicators to measure the 26 provinces, showed that the difference between the two was small, and the *p*-value of the homogeneity of variance test was greater than 0.05. At the same time, the decentralization levels in the north and south of the Huai River are 0.449, which does not reject the original hypothesis that there is no significant difference. That is, there is no significant difference in the level of governance decentralization between the north and the south. However, there is a significant difference in governance input between the north and south of the Huai River. The governance investment level is higher in the north of the Huai River, with an ultimately higher lever of governance uncertainty against the background of decentralized prevention and control. The south of the Huai River is dominated by light industries such as electronics and clothing, while the north of the Huai River is dominated by heavy industries such as steel and non-ferrous metals. These factors, combined with the APHD, significantly inhibit economic growth. Further, not only does the APHD in the north of Huai River have a larger impact on production and the lives of residents, but also the environmental governance input has a larger impact on the development of heavy industry in the region, which significantly inhibits economic growth. These results show that the northern Huai River needs to further improve the use of investment funds for governance.

Additionally, according to Zhang and Wang (2020) [6], we calculated the self-defense ability of different regions (see their original introduction for the measurement details) and divided the samples into three categories by means of the mean clustering method: “<0.345,” “0.345~0.487,” and “>0.487,” which respectively represent low, medium, and high self-defense ability in different regions. As shown in Table 4 (see Column (18)~(20)), governance uncertainty in regions with low and medium levels of self-defense ability has a significant negative impact on economic growth, with the influence elasticity ranging from 1.3% to 1.8%.This result indicates that improving regional self-defense abilities can effectively alleviate the restraining influence of governance uncertainty on economic growth.

### 4.4. The Threshold Effect Results

**Figure 4:**
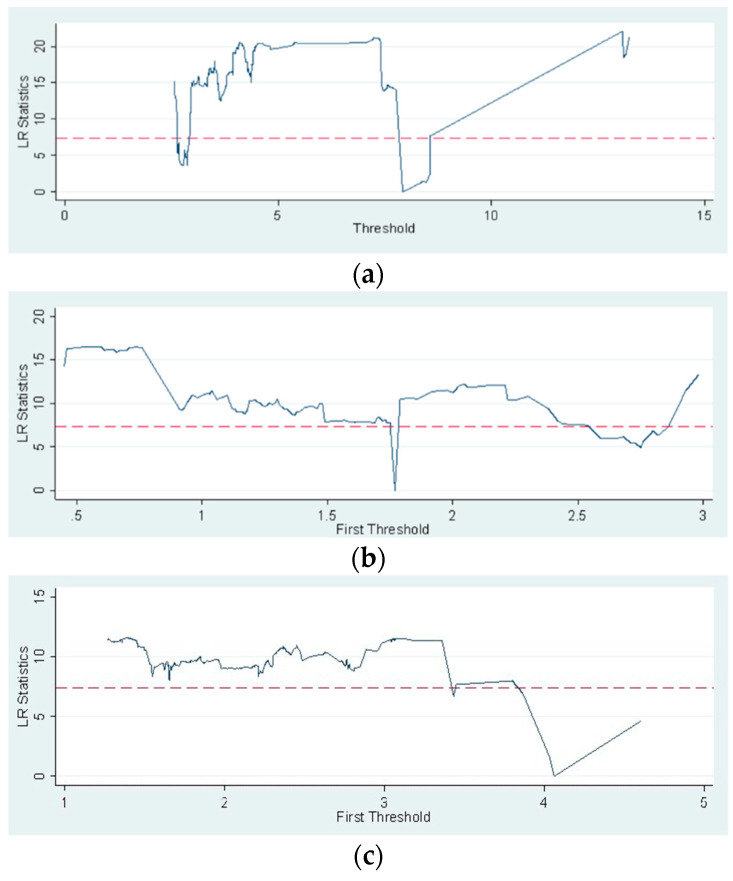
The LR statistics value trend estimated for the threshold variable. (**a**) Decentralization level; (**b**) Governance input level; (**c**) APHD level. And, the dashed line is the critical value of 7.35.

We selected three factors closely related to the governance uncertainty: the level of decentralization of prevention and control, the level of governance input, and the level of APHD for threshold effect analysis. Since it is of little significance to choose governance uncertainty as the threshold variable, we pay more attention to how to control the factors that affect governance uncertainty, such as the level of decentralized prevention and control and the level of governance input. According to the estimation results in Table 5, the three threshold variables all significantly have a threshold effect at the 10% confidence level. As shown in Figure 4, the LR values of the first threshold value of the three variables are far less than the critical value of 7.35 (the critical value at the 5% significance level), while the second threshold values do not meet the conditions, indicating that the threshold number identification is more reliable. Space being limited, the main chart is shown below; other content is welcome upon request. Table 6 shows that the single threshold values for decentralization level, governance input level, and APHD level are 7916, 1770, and 4061, respectively. 

In Column (21) of Table 7, we can see that the interaction between governance uncertainty and pollution-related health damage can significantly inhibit economic growth under the condition of a low control decentralization level (level value <7.916). Under other conditions, economic growth decreases by 0.4% on average for each unit increase in the interaction term. Using the sample descriptive statistical analysis, we found that the lower the decentralization level of prevention and control in the region, the lower the economic growth level, the higher the proportion of secondary industry, and the higher the proportion of environmental governance input in GDP. This result shows that these regions are paying more attention to environmental pollution control at the expense of greater economic growth interests. In addition, the industrial structure of these regions is relatively homogenous, and the development of green economic systems is not yet mature, which ultimately leads to the uncertainty imposed on air pollution and health problems, significantly inhibiting economic growth. Meanwhile, a lower decentralization level is easy to generate a higher incentive space for rent-seeking behavior of economic interests due to excessive centralization, which is easy to cause pollution rebound and commons tragedy. This will further exacerbate health damage and inhibit economic growth. Therefore, in the case of a low decentralization level, the interaction between governance uncertainty and APHD can significantly inhibit economic growth. 

In Column (22), when the input of governance of gdp is greater than 1.77%, the interaction between governance uncertainty and aphd will significantly inhibit economic growth. When other conditions are given, economic growth decreases by 0.5% on average for each unit increase in the interaction term. Because regions with large investments in environmental pollution control pay more attention to environmental pollution control, the governance uncertainty caused by the policies’ implementation will significantly reduce economic growth. This further confirms the characteristics of regions with a low decentralization level of pollution prevention and control, indicating that regions tend to spend more on governance and that their governance uncertainty acting on APHD can significantly hinder economic growth.

In Column (23), when the APHD level is less than 4.061, the interaction between governance uncertainty and APHD will significantly inhibit economic growth. When other conditions are given, economic growth decreases by 0.4% on average for each unit increase in the interaction term. These results suggest that when the level of APHD is low, the uncertainty created by haphazard policies can significantly inhibit economic growth. It can be seen that local governments need scientific assessment before implementing governance policies. Therefore, under the condition of a certain APHD level, a reasonable setting of the decentralization level of prevention and control and pollution control investment, that is, when the decentralization level of prevention and control is higher than 7.916 and the pollution control investment of GDP is lower than 1.77%, can effectively resist the negative impact of control uncertainty on economic growth.

## 5. Discussion

Based on China’s typical decentralized governance system, this paper measures the governance uncertainty index considering the governance input cost of pollution and health problems and the decentralized governance methods (including economic decentralization, administrative decentralization, and fiscal decentralization). This is different from previous studies. Current research on policy uncertainty mainly involves economic policy uncertainty, fiscal policy uncertainty, etc. [38,78,79,80]. Most of them make use of the vocabulary text and other uncertain words involved in the policy. For example, the economic policy uncertainty index, as currently measured, mainly includes three quantitative components: newspaper reports on policy-related economic uncertainty, the number of federal tax provisions that will expire in the next few years, and the divergence among economic forecasters [67]. There are studies that also used the information from several local newspapers to measure the monthly economic uncertainty index based on China’s national conditions [81]. Although the uncertainty index of these measures is relatively comprehensive, scientific, and widely applied, it has two limitations: First, the measurement of China is not specific to each provincial level or smaller level, so scholars cannot use panel samples for analysis. Second, it only focuses on the measurement of economic policy uncertainty and lacks the measurement of general governance policy implementation uncertainty. Therefore, the governance uncertainty index innovatively designed and measured in this paper has the feature of panel change and can reflect the uncertainty of governance policy implementation in the decentralized governance system. This is helpful for scholars to learn from and carry out more research.

Then, based on the measurement of relevant indexes, this paper analyzes the relationship among the APHD, governance uncertainty, and economic growth from the perspective of the implementation process of comprehensive governance policy. The results show that: The APHD seriously hinders economic growth. The influence elasticity was −1.233%. However, the external cost of air pollution in China accounts for about 1−8% of GDP [25], and the elasticity of impact is within this range, indicating that the estimate is relatively reliable. At the same time, the governance uncertainty promotes economic growth to a certain extent, but when it acts on the APHD, it will hinder economic growth. The hindrance elasticity of the interaction term is −1.3%, which is greater than the influence elasticity of −1.233% when there is no interaction term. This suggests that decentralization of governance in the process of air pollution control (resulting in uncertainty) has a significant negative impact on economic growth [15,37]. When the governance power is delegated to the county level, the economic negative effect of the interaction between the governance uncertainty and the APHD is smaller, and the average impact elasticity is 0.2%~0.3%. This proves that decentralization of environmental governance to the county level is more conducive to environmental governance and economic growth than decentralization to the municipal level [76]. It is imperative for the government to take joint prevention and treatment measures. However, the relationship among the three is rarely studied at the same time. Previous studies focused more on the impact of environmental pollution or air pollution on economic growth, and their conclusions were mixed [16,17,21]. They also lacked pertinence in assessing specific impact paths. We measure the APHD with reference to the existing study [66], evaluate the relationship between APHD and economic growth, and give a more accurate effect. This is mainly because there are many ways that air pollution can affect economic growth, and the results are not convincing without locking in the specific impact path. However, the health damage effect from air pollution is more extensive and common, and it is an important path to affect economic growth. Meanwhile, healthy development is also an important issue for the Chinese government to focus when building a “healthy China.” Therefore, it is important to accurately estimate the impact of air pollution on economic growth. Additionally, most scholars have studied the relationship between specific policy uncertainty and economic growth in China and believe that economic policy uncertainty or fiscal policy uncertainty has a close relationship with economic growth over a certain period of time [82,83,84]. However, their assessment of governance uncertainty is not comprehensive, let alone from the perspective of the policy implementation process, and the reliability of the conclusions is questionable.

However, there are several limitations to our study: First, the study period is 2007–2015. Due to the interference of policy effects and the limitation of data, we do not extend the data to recent years, and the evaluation results may be biased. Secondly, the measurement of the governance uncertainty index mainly comprehensively considers factors such as governance input cost and governance mode and ignores the consideration of other factors affecting the uncertainty, which we will explore above in the future.

## 6. Conclusions and Policy Implications

### 6.1. Conclusions

This article adopts China’s inter-provincial panel samples to measure the uncertainty caused by the implementation of relevant policies to solve environmental and health problems based on China’s special decentralized governance system and analyzes the relationship between the APHD, governance uncertainty, and economic growth from the perspective of the assessment of the governance process. The main conclusions are as follows:

1. The APHD inhibits economic growth, and the governance uncertainty can promote economic growth, but the governance uncertainty will aggravate the inhibiting effect of the APHD on economic growth. When controlling other conditions, for each 1% increase in the interaction item of APHD and governance uncertainty, economic growth will drop by 1.3%. The robustness tests confirm the reliability of these conclusions. Specifically, when the governance power is delegated to the municipal level, the interaction between the governance uncertainty constructed by expenditure fiscal decentralization and APHD has a larger negative economic effect. While the governance power is delegated to the county level, the interaction between the governance uncertainty constructed by income fiscal decentralization and APHD has a smaller negative economic effect.
2. The negative relationship between the APHD and economic growth has a spatial characteristic due to heterogeneous conditions. Specifically, there is a negative impact that has increased successively in the east, middle, and west regions of China. Moreover, the negative effect is significant in the north of the Huai River with medium and low self-defense capabilities. This indicates that the mismatch between governance input and resource endowment will exacerbate this negative effect, and the negative effect caused by the level of decentralization is vulnerable to the interference of economic level, industrial structure, and other factors, but the improvement of self-defense capability can reduce this negative effect.
3. Under the conditions of a low decentralization level, a high governance input level, and a low APHD level, there is a single threshold value of significant influence. When the APHD level reaches a certain threshold value (such as 4.061), and the combined boundary value of the decentralization level and the governance input is slightly higher than 7.916 and lower than 1.77% of GDP, can the government effectively resist the negative impact of governance uncertainty on economic growth.

### 6.2. Policy Implications

Based on the issues above, the policy implications are as follows:

1. The government should work hard to reduce air pollution and its damaging effects on health. The government can vigorously develop the green and health industries, raise residents’ awareness of healthy living, and improve regional self-defense abilities, so as to reduce the level of health damage caused by air pollution. Special attention should be paid to areas north of the Huai River or areas with low self-control abilities.
2. The government should reasonably increase investment in governance, preferably below 1.77% of GDP. The investment in environmental governance should be continuously increased, but blindly increasing investment in environmental governance is easy to induce rent-seeking behavior in the process of implementation. Meanwhile, in regions with a slower pace of industrial restructuring, overinvestment in governance will directly inhibit economic growth and lead to a waste of resources disproportionate to pollution levels. Therefore, the government should fully examine the actual endowment conditions, pay special attention to the western and central regions, and reasonably increase the governance investment.
3. The government should reasonably evaluate the decentralization of governance and further improve the performance of joint prevention and treatment. The greater the level of governance decentralization, the more likely it is to lead to inadequate and uncoordinated use of governance power and easily aggravate the governance uncertainty. Although the governance uncertainty is relatively small when the governance decentralization level is too low, due to the interference of factors such as the original economic level, it will aggravate the inhibiting effect of APHD on economic growth. Therefore, the government should reasonably evaluate the spatial scope, specific work content, and implementation standards of joint prevention and control according to the actual situation of the original economic level and industrial structure of each region to ensure the full and coordinated use of governance power and reduce the adverse impact.

## Figures and Tables

**Table 1:** The descriptive statistics of the variables.

Var.	Name	Description	Mean	Std. Dev.	Min	Max
pergdp	economic level	Per capita GDP (yuan/person)	35,552.500	19,951	10,346	106,184
h	APHD	A composite index of different health endpoints associated with different air pollution	2.053	0.824	1.085	7.389
proex	Investment in pollution control	Investment in environmental pollution control as a share of GDP (%)	1.391	0.633	0.400	3.760
bxt1	Prevention and control decentralization index 1	The uncoordinated comprehensive index is constructed by the expenditure fiscal power, the municipal territorial jurisdiction, and the economic development right under the Gini coefficient	0.913	0.354	0.413	2.247
bxt2	Prevention and control decentralization index 2	The uncoordinated comprehensive index constructed by income fiscal rights, county territorial jurisdiction, and economic development rights under range representation	4.322	2.269	1.704	14.122
bp	Governance uncertainty index 1	Governance uncertainty index at the municipal level constructed by *proex* and *bxt1*	1.255	0.721	0.333	4.817
bpp	Governance uncertainty index 2	Governance uncertainty index at county level constructed by *proex* and *bxt2*	5.918	3.567	1.408	21.746
k	Stock of capital per capita	Stock of capital per capita (ten thousand yuan/person)	11.299	6.526	1.691	32.982
iemport	Regional trade openness	The ratio of total imports and exports to GDP (%)	0.160	0.235	0.0003	1.101
pere	Per capita capital level	Average years of education (Year/person)	10.954	1.127	8.267	14.610
ur	Development of urbanization	Urbanization rate (%)	2.048	3.248	0.075	15.854
secr	The industrial structure	Ratio of output value of secondary industry to GDP (%)	48.633	7.081	19.738	61.500

Note: The sample size is 234.

**Table 2:** Results of step-to-step regression estimation of panel fixed effect model.

Var.	(1)	(2)	(3)	(5)	(6)	(7)	(8)	(9)
h	−0.046 ***	−0.045 ***	−0.037 ***	−0.020 ***	−0.021 ***	−0.019 ***	−0.020 ***	−0.012 ***
	(−4.90)	(−8.09)	(−5.31)	(−4.39)	(−4.69)	(−4.32)	(−4.49)	(−3.08)
bp		0.023 ***	0.030 **	0.016 ***	0.016 ***	0.015 **	0.017 ***	0.012 **
		(2.81)	(2.63)	(2.61)	(2.67)	(2.57)	(2.84)	(2.21)
hbp			−0.024 *	−0.011 **	−0.010 **	−0.011 **	−0.01 1 **	−0.013 ***
			(−1.77)	(−2.36)	(−2.20)	(−2.54)	(−2.43)	(−3.42)
lnk				0.231 ***	0.215 ***	0.206 ***	0.215 ***	0.193 ***
				(12.24)	(10.68)	(10.31)	(10.78)	(10.74)
iemport					0.091 **	0.090 **	0.151 ***	0.102 **
					(2.29)	(2.29)	(3.37)	(2.54)
pere						0.027 ***	0.028 ***	0.014 *
						(2.94)	(3.08)	(1.70)
ur							0.037 ***	0.037 ***
							(2.68)	(3.03)
secr								0.006 ***
								(7.47)
Constant	−188.194 ***	−185.902 ***	−186.461 ***	−104.660 ***	−112.549 ***	−105.008 ***	−97.728 ***	−116.10 1 ***
	(−32.65)	(−71.30)	(−34.02)	(−15.06)	(−14.63)	(−13.17)	(−11.76)	(−14.96)
Individual effect	*Y*	*Y*	*Y*	*Y*	*Y*	*Y*	*Y*	*Y*
Time effec	*Y*	*Y*	*Y*	*Y*	*Y*	*Y*	*Y*	*Y*
Observations	234	234	234	234	234	234	234	234
r2_a	0.968	0.966	0.972	0.982	0.982	0.983	0.983	0.987
F	634.7	2190	390.2	2499	2127	1893	1709	1941

Note: *, **, *** refers to significance at the level of 10%, 5%, and 1%, respectively. The number in parentheses is t value. The same below.

**Table 3:** The robustness test results of the baseline model.

Var.	(10)	(11)	(12)
h	−0.013 ***		
	(−3.10)		
bpp	0.002 *		
	(1.82)		
hbpp	−0.002 ***		
	(−2.97)		
L.h		−0.016 ***	−0.016 ***
		(−4.33)	(−4.29)
L.bp		0.005	
		(1.17)	
L.hbp		−0.013 ***	
		(−3.61)	
L.bpp			0.001
			(1.15)
L.hbpp			−0.003 ***
			(−3.54)
Control variables	*Y*	*Y*	*Y*
Individual effect	*Y*	*Y*	*Y*
Time effect	*Y*	*Y*	*Y*
Observations	234	208	208
r2_a	0.987	0.988	0.988
F	1905	1853	1847

Note: *, *** refers to significance at the level of 10% and 1%, respectively.

**Table 4:** Estimated results of heterogeneous conditions.

Var.	(13)	(14)	(15)	(16)	(17)	(18)	(19)	(20)
	The Eastern Region	The Central Region	The Western Region	North of Huai River	South of Huai River	The Low Self-Defense Capability Region	The Low Self-Defense Capability Region	The Low Self-Defense Capability Region
hbp	−0.008 *	−0.019 *	−0.079 ***	−0.016 **	0.013	−0.013 ***	−0.018 **	0.005
	(−1.67)	(−1.78)	(−2.92)	(−2.20)	(1.47)	(−3.30)	(−2.05)	(0.39)
Control variables	Y	Y	Y	Y	Y	Y	Y	Y
Individual effect	Y	Y	Y	Y	Y	Y	Y	Y
Time effect	Y	Y	Y	Y	Y	Y	Y	Y
Observations	90	72	72	117	117	63	90	81
r2_a	0.987	0.993	—	0.987	0.994	0.993	0.993	0.993
F	750.900	1119	2476.420	553.200	3803	1016	1380	1294

Note: *, **, *** indicates statistically significant at the 1%, 5%, and 10% confident level respectively. The regions with low, medium, and high self-defense capability are: (Beijing, Tianjin, Shanghai, Jiangsu, Hubei, Hunan, and Guangdong), (Jilin, Heilongjiang, Zhejiang, Anhui, Henan, Chongqing, Sichuan, Shaanxi, Gansu, and Ningxia), and (Hebei, Shanxi, Inner Mongolia, Liaoning, Fujian, Jiangxi, Shandong, Guangxi, and Yunnan).

**Table 5:** Identification results by threshold number.

Threshold Variable	Threshold	RSS	MSE	Fstat	Prob	Crit10	Crit5	Crit1
Level of decentralization	Single	0.1561	0.0007	24.02	0.0500	19.3091	23.8846	35.8206
	Double	0.1492	0.0007	10.41	0.3467	17.3579	22.9535	40.9040
	Triple	0.1382	0.0006	17.95	0.3633	35.3862	43.5081	58.8610
Level of governance input	Single	0.1596	0.0007	18.61	0.0367	13.1519	17.5744	23.4323
	Double	0.1557	0.0007	5.65	0.4700	13.1583	15.9310	26.4815
	Triple	0.1530	0.0007	3.94	0.7167	10.9292	13.4010	20.8247
Level of APHD	Single	0.1631	0.0007	13.39	0.0567	10.0695	13.5312	21.5948
	Double	0.1591	0.0007	5.60	0.3767	10.4659	13.7275	29.5553
	Triple	0.1582	0.0007	1.34	0.9733	11.5220	14.2227	36.7065

**Table 6:** The estimation of single threshold point.

Threshold Variable	Model	Threshold	Lower	Upper
Level of decentralization	Th-1	7.9157	7.7575	8.3251
Level of governance input	Th-1	1.7700	1.7500	2.2500
Level of APHD	Th-1	4.0608	3.9150	4.5980

**Table 7:** The impact effect estimation of threshold points.

Var.	(21)	(22)	(23)
	Threshold variable: Level of Decentralization	Threshold Variable: Level of Governance Input	Threshold Variable: Level of APHD
h	−0.015 ***	−0.011 ***	−0.015 ***
	(−3.69)	(−2.69)	(−3.84)
bpp	0.002	0.003 **	0.002 *
	(1.65)	(2.15)	(1.78)
0b._cat#c.hbpp	−0.004 ***	−0.000	−0.004 ***
	(−4.06)	(−0.34)	(−4.35)
1._cat#c.hbpp	−0.000	−0.005 ***	−0.000
	(−0.46)	(−5.04)	(−0.24)
Control variables	Y	Y	Y
Observations	234	234	234
r2_a	0.987	0.988	0.987
F	1772	1849	1809

Note: *, **, *** refers to significance at the level of 10%, 5%, and 1%, respectively.

## Data Availability

Data is available on request.
